# Primary cutaneous malignant granular cell tumor: a case report in China and review of the literature

**DOI:** 10.1186/s13000-015-0357-2

**Published:** 2015-07-19

**Authors:** Ting-ting Liu, Yang Han, Song Zheng, Bo Li, Yu-qi Liu, Yi-xian Chen, Yong-feng Liu, En-hua Wang

**Affiliations:** Department of General Surgery, First Affiliated Hospital of China Medical University, Shenyang, China; Department of Pathology, First Affiliated Hospital of China Medical University, Shenyang, China; Department of Dermatology & STD, First Affiliated Hospital of China Medical University, Shenyang, China; Graduate School of China Medical University, Shenyang, China

**Keywords:** Malignant granular cell tumor, Cutaneous

## Abstract

Granular cell tumor is rare and accounts for approximately 0.5 % of all soft tissue tumors. The malignant granular cell tumor, especially cutaneous malignant granular cell tumor is extremely rare. The present case is the first patient of primary cutaneous malignant granular cell tumor reported from China in English. A review of the literature is performed, and the presentation, position, pathological diagnosis, treatment and prognosis of the patients with cutaneous malignant granular cell tumor of the reported cases before is analysed.

## Background

Granular cell tumor is rare and accounts for approximately 0.5 % of all soft tissue tumors [1]. The frequent locations of the tumor are tongue (40 %), breast (15 %), respiratory tract (10 %) and oesophagus (2 %). Granular cell tumor can be multicentric (5 % to 14 % of cases) [2]. The malignant granular cell tumors (MGCT) are extremely rare representing less than 0.5 % to 2 % of all granular cell tumors and more likely to attack blacks than whites and are two times more likely to occur in females than males [3]. Both benign and malignant granular cell tumors have been found in a wide variety of locations, including lung, heart, pelvis, bladder, vulva, abdominal wall, and esophagus [4–12]. Local recurrence and metastasis of MGCT is potentially higher with poor prognosis than the benign counterparts. MGCT also typically develop in the lower extremity, often the thigh, whereas the benign tumors more commonly occur in the head and neck, most commonly the tongue. The most common metastasis sites are regional lymph nodes, lungs, and bones [13]. Primary cutaneous malignant granular cell tumor is very rare, here we report a case and review literature.

### Case presentation Clinical history

A 66-year-old man presented with a mass in the skin of the right abdominal wall, which was first noted approximately three years ago, however the size of the mass has been increased gradually over the past six months without pain. On examination, a well-localized, about 3.0 cm in diameter soft tissue mass was clinically located in the skin and bulged from the right abdomen wall. The patient was otherwise well, with no other medical conditions. There was no family history of any malignancy or cutaneous lesions. Local excision was performed for the primary tumor. No dissection of lymph nodes and adjuvant treatment was performed. The patient is well, without recurrence one year after surgery.

### Pathology

#### Gross

**Fig 1:**
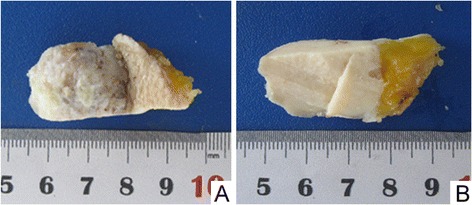
**a**, The mass bulged from the skin with a rough surface, ulceration and necrosis. The mass was well circumscribed with 3.0 cm in diameter and bulged from the skin with 2.8 cm high; **b**, The section was firm and gray white with toughness. The normal structure of the skin disappeared and the tumor invasion to the subcutaneous fat layer

The mass was firm and well circumscribed with 3.0 cm in diameter and bulge from the skin with 2.8 cm high. The surface of the mass was rough with ulceration and the section was gray white with toughness. The tumor was located in the dermis and the subcutis and invaded to the subcutaneous fat layer with the normal structure of the skin disappeared (Fig. 1a and b).

#### Histology and Immunohistochemistry

**Fig 2:**
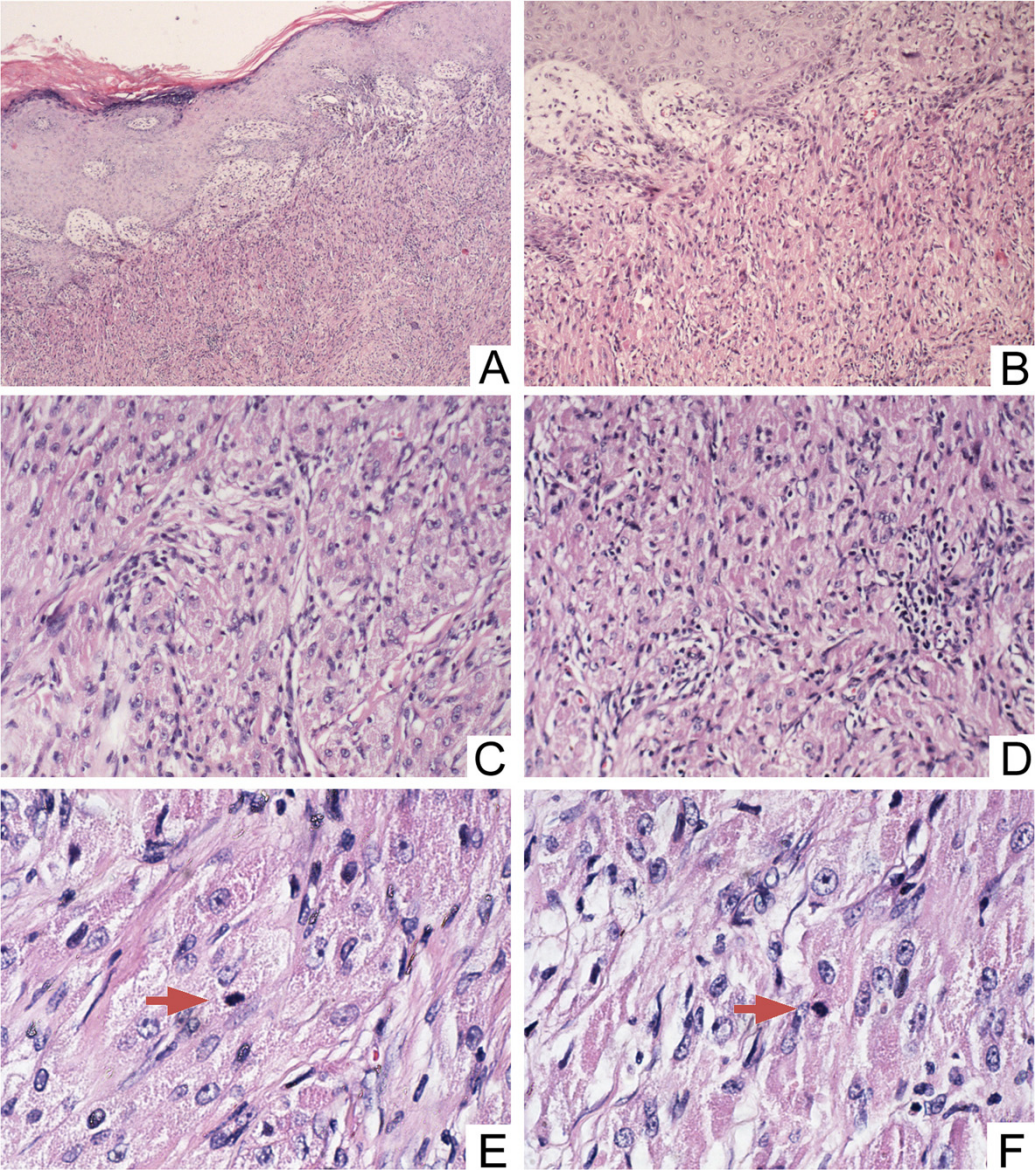
**a** and **b**, the tumor was composed of aggregates and sheets of intermediate size spindle and polyhedral cells that had granular eosinophilic cytoplasm, A, H-E stain, 40× and B, H-E stain, 100×; **c** and **d**, H-E stain, 200×; **e** and **f**, high nucleocytoplasmic ratio, vesicular nuclei with prominent nucleoli, the arrows show mitoses, H-E stain, 400 ×

**Fig 3:**
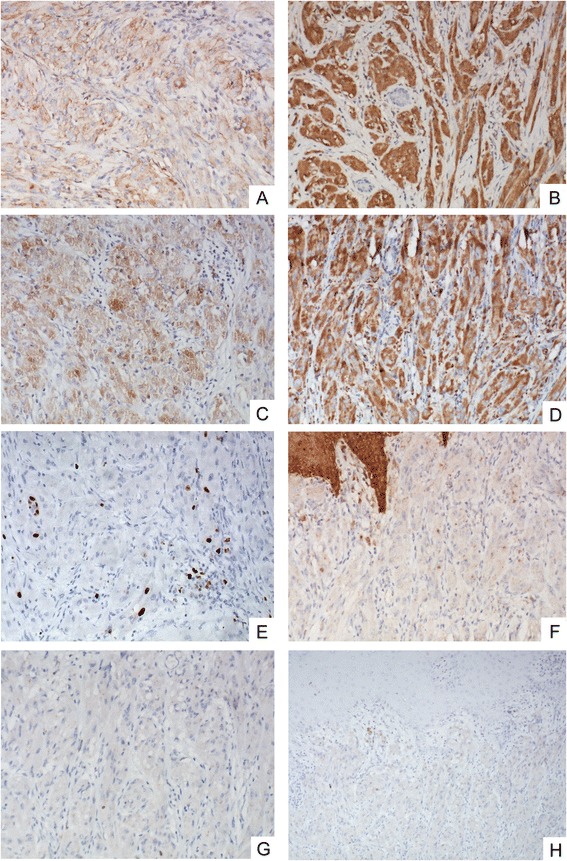
**a**, vimentin, 200×; **b**, NSE, 200×; **c**, S-100, 200×; **d**, CD68, 200×; **e**, Ki-67, 200×; **f**, CK, 200×; **g**, Desmin, 200×; **h**, GFAP, 100 ×

The tumor and the other abscised tissues were fixed in 10 % formalin and embedded in paraffin. Several 4-μm sections were cut from each paraffin block, and one was stained with HE (hematoxylin and eosin), the others were stained with IHC (immunohistochemistry). Immunohistochemical staining was performed using the streptavidin-peroxidase system (Ultrasensitive; Mai Xin Inc., Fuzhou, China) according to the manufacturer’s instruction. Commercially available prediluted monoclonal antibodies against the following antigens were employed: vimentin, S100, NSE and CD68, cytokeratin (with CAM 5.2 and KL-1), actin (smooth muscle), desmin, GFAP, EMA, CD34, HMB45 and NF-LH(all Thermo Fisher Scientific Inc., Fremont, CA, USA). Histologically, the tumor was composed of aggregates and sheets of intermediate size spindle and polyhedral cells that had granular eosinophilic cytoplasm. The nuclei were vesicular with prominent nucleoli. Mitoses were numerous with 1 mitoses per 10 high-power fields. High nucleocytoplasmic ratio was present. No definite necrosis (Fig. 2). Immunohistochemistry was positive for vimentin, S100, NSE and CD68, and negative for cytokeratin (with CAM 5.2 and KL-1), actin (smooth muscle), desmin, GFAP, EMA, CD34, HMB45 and NF-LH. The proliferation rate assessed by the Ki-67 (MIB-1) stain was about 10 % (Fig. 3).

## Conclusions

Histologically, the tumor was composed of aggregates and sheets of intermediate size spindle and polyhedral cells that had granular eosinophilic cytoplasm. Mitoses were numerous with 1 mitoses per 10 high-power fields. Immunohistochemistry was positive for vimentin, S100, NSE and CD68, and negative for cytokeratin, actin (smooth muscle), desmin, GFAP, EMA, CD34, HMB45 and NF-LH. Ki-67 (MIB-1) stain was about 10 %. The diagnosis of primary cutaneous malignant granular cell tumor was made.

## Discussion

Granular cell tumor was first described in German literature in 1926 and named myoblastoma, with believed to be of myogenic origin because of its origin from the tongue muscle [23]. The tumor was thought to have a neural origin because it was assumed to be from the radial and sciatic nerves within the extremities in 1960s [14]. Recent decades, Schwannian features have been documented ultrastructurally and immunohistochemically [15–19]. This rare tumor can affect various regions of the body, but more frequently found in the head and neck commonly involving the tongue. Primary cutaneous malignant granular cell tumor is very rare.

In China, there is only one case report of primary cutaneous malignant granular cell tumor in Chinese. A 55-year-old women, a farmer, complained of a hard nodule, which diameter was 2.5 cm, on the back for about 1 year. The patient had a good condition after surgery and still under follow up since the paper was published [24].

Here, we present a Chinese patient of primary malignant granular cell tumor of cutaneous. The tumor was firm and well circumscribed with 3.0 cm in diameter and bulge from the skin with 2.8 cm high. The surface of the mass was rough with ulceration and the section was gray white with toughness. Histologically, the tumor was composed of aggregates and sheets of intermediate size spindle and polyhedral cells that had granular eosinophilic cytoplasm. The nuclei were vesicular with prominent nucleoli. Immunohistochemistry was positive for vimentin, S100, NSE and CD68. The proliferation rate assessed by the Ki-67 (MIB-1) stain was about 10 %.

Granular cell tumor usually presents with a painless mass occurring most during 30–50 years old. Women are more commonly affected with a ratio of 1.8-2.9:1 to men. The best imaging modality for the characterization of this tumor is magnetic resonance imaging (MRI)[14]. Clinical findings for prediction of malignancy includes large tumor size (>5 cm), older age, female gender, oval or round shape, deep location (intramuscular), occurrence in the lower extremities, rapid recent growth after an extended period, and local recurrence [20]. The differential diagnosis of MGCT includes renal cell carcinoma, rhabdomyosarcoma, and alveolar soft part sarcoma, ect. However, the diagnosis usually can be confirmed by the histomorphology and immunohistochemical profile.

Fanburg-Smith et al. reported histologic criteria for MGCT by analyzing the clinical and histologic data of 73 cases of granular cell tumors. According to their report, the six features include necrosis, spindling, vesicular nuclei with large nucleoli, increased mitotic activity (>2 mitoses/10 high-power fields at 200× magnification), high nuclear to cytoplasmic (N:C) ratio, and pleomorphism. Neoplasms that met three or more of these criteria were classified as histologically malignant, which may result in death in 40 % of cases because of the high chance of local recurrence and metastasis. Those that met one or two criteria were classified as atypical; and those that displayed only focal pleomorphism but fulfilled none of the other criteria were classified as benign with no metastasis or local recurrence after adequate resection. Poor prognostic factors associated with MGCT include large tumor size, older patient age, increased mitotic activity and Ki-67 greater than 10 % [21].

Surgical excision with a clean margin is the best treatment for this tumor although it is not always possible because of lacking a complete capsule. In addition to the incidence of recurrence and metastasis and the rapidity of tumor growth, a second operation and postoperative chemotherapy and/or radiation should be considered.

Local recurrence and metastasis are relatively common in MGCT, with 32 % rate of recurrence and 50 % metastasis in the Fanburg-Smith analysis [21]. Distant and lymph node metastases are common, presenting between 3 and 37 months after initial diagnosis, and distal metastases often occur in the lung, liver and bone [13, 20–22]. Thus, a sentinel lymph node biopsy during the initial surgical resection should be necessary.

## Consent

Written informed consent was obtained from the patient for publication of this Case report and any accompanying images. A copy of the written consent is available for review by the Editor-in-Chief of this journal.
